# The emerging roles of the macular pigment carotenoids throughout the lifespan and in prenatal supplementation

**DOI:** 10.1194/jlr.TR120000956

**Published:** 2021-02-06

**Authors:** Paul S. Bernstein, Ranganathan Arunkumar

**Affiliations:** 1 Department of Ophthalmology and Visual Science, Moran Eye Center, University of Utah School of Medicine, Salt Lake City, UT, USA

**Keywords:** age-related macular degeneration, antioxidant, lutein, *meso*-zeaxanthin, nutrition, ocular health, zeaxanthin, ocular development, retina, AMD, age-related macular degeneration, BCO1, β-caroteneoxygenase 1, BCO2, β-carotene-oxygenase 2, GSTP1, glutathione S-transferase P1, L, lutein, MacTel, macular telangiectasia type 2, MP, macular pigment, MZ, meso-zeaxanthin, ROP, retinopathy of prematurity, RPE, retinal pigment epithelium, RPE65, retinoid isomerase, RRS, resonance Raman spectroscopy, Z, zeaxanthin

## Abstract

Since the publication of the Age-Related Eye Disease Study 2 (AREDS2) in 2013, the macular pigment carotenoids lutein (L) and zeaxanthin (Z) have become well known to both the eye care community and the public. It is a fascinating aspect of evolution that primates have repurposed photoprotective pigments and binding proteins from plants and insects to protect and enhance visual acuity. Moreover, utilization of these plant-derived nutrients has been widely embraced for preventing vision loss from age-related macular degeneration. More recently, there has been growing awareness that these nutrients can also play a role in improving visual performance in adults. On the other hand, the potential benefits of L and Z supplementation at very young ages have been underappreciated. In this review, we examine the biochemical mechanisms and supportive data for L and Z supplementation throughout the lifespan, with particular emphasis on prenatal supplementation. We propose that prenatal nutritional recommendations may aim at improving maternal and infant carotenoid status. Prenatal supplementation with L and Z might enhance infant visual development and performance and may even prevent retinopathy of prematurity, possibilities that should be examined in future clinical studies.

## Biochemical and anatomical background

**Fig. 1:**
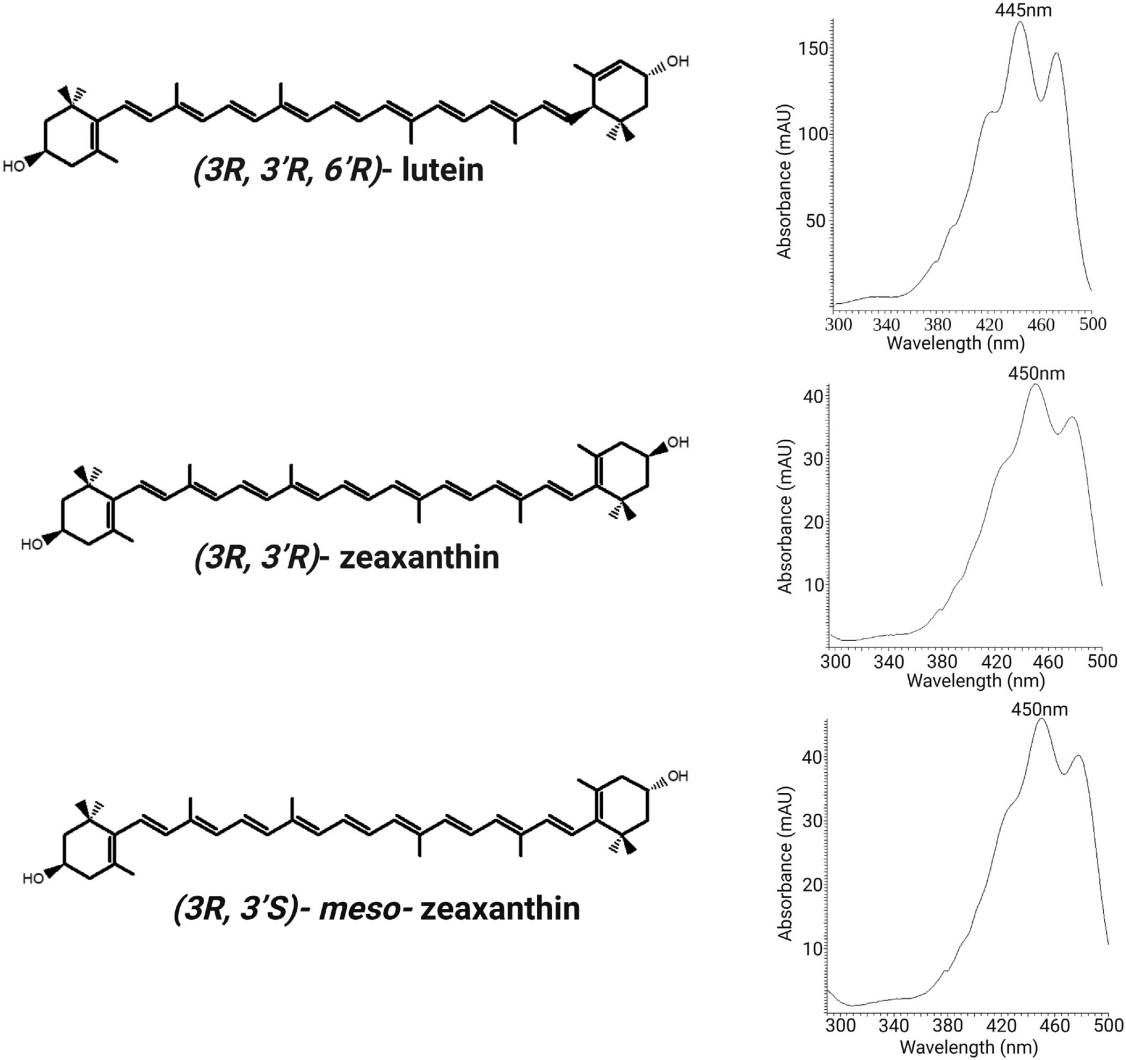
Chemical structures and absorption spectra of the MP carotenoids: L, Z, and MZ.

Lutein (L) and zeaxanthin (Z) are plant-derived pigments frequently consumed in a normal human diet that are naturally concentrated in the macula lutea (yellow spot) of the human eye where they are major components of the macular pigment (MP) (1, 2). They are lipophilic members of the xanthophyll carotenoid class that share a common C_40_H_56_O_2_ molecular formula (Fig. 1). They have a rigid 22-carbon isoprenoid backbone with nine conjugated C=C double bonds. Ionone rings connect to each end of the backbone, two β rings in the case of Z, or one epsilon and one β ring in the case of L (3). This increases C=C double bond conjugation to 10 for L and to 11 for Z and shifts the visible absorbance maximum in methanol for L to 445 nm and to 450 nm for Z. Each of the ionone rings is hydroxylated at the *3*- or *3′*-position. These hydroxyl groups modestly increase aqueous solubility of these xanthophylls relative to their hydrocarbon congeners, such as β-carotene, and render their central cleavage products devoid of any vitamin A activity. The hydroxyl groups also generate chiral centers at each *3*- and *3*′-position. Dietary and ocular Ls are exclusively the *(3R,3′R,6′R)*-L isomer. Dietary Z is almost exclusively the *(3R,3′R)*-Z isomer, but primate and avian ocular tissues have an additional nondietary stereoisomer, *(3R,3′S)-meso*-Z (MZ) (3).

Vertebrates cannot synthesize any carotenoids de novo and must obtain them exclusively from the diet (4). Americans typically consume 1–2 mg of L per day, primarily from green leafy vegetables, some orange and yellow fruits and vegetables, and egg yolk. Z is less common in the American diet, with typical daily intakes in the 0.2–0.4 mg range from a narrower range of foods such as corn, persimmons, orange peppers, and egg yolk. MZ is found only in rarely eaten foods such as eyeballs, fish skin, and turtle fat (5). While typical ranges of dietary intake are provided above, there is considerable variation in the population; some people avoid fruits and vegetables and thus consume very low levels of the MP carotenoids, while vegetarians can easily consume 5–10 times the average American daily intake. Supplements containing L, Z, and MZ are widely available in many combinations and dosages, commonly at a 5–10 times dose relative to average American intake. The MP carotenoids are designated by the US Food and Drug Administration as generally recognized as safe for human consumption at total doses in the 20 mg/day range (6). Only rare cases of crystalline deposition of carotenoids in the macula have been reported in association with excessive L or Z supplementation (7).

**Fig. 2:**
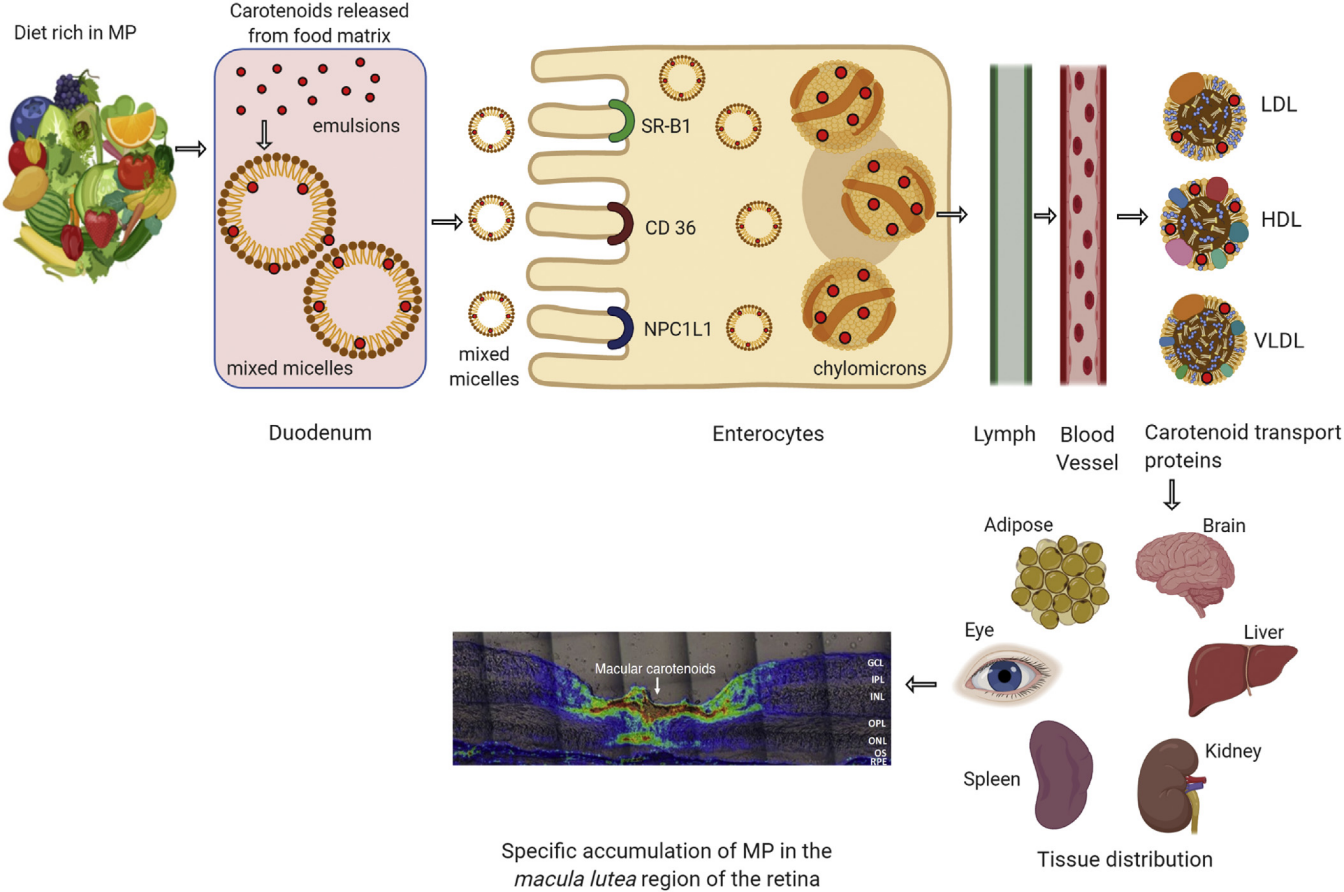
Possible pathways for MP carotenoid uptake, transport, and accumulation in the human retina. The red dots denote the MP carotenoids: L, Z, and MZ.

Dietary or supplemental MP carotenoids can be in an unesterified (free) form or conjugated to long-chain fatty acids, but such esters are rapidly cleaved in the lumen of the gut (Fig. 2) (8). They are absorbed through the epithelium of the intestine and packaged into chylomicrons and initially delivered to the liver where they are repackaged onto lipoproteins for delivery to the peripheral tissues (8). Among mammals, only primates avidly take up xanthophyll carotenoids from the diet, while most other mammals, and especially rodents, have two very active β-carotene-oxygenase enzymes (BCO1 and BCO2) that cleave ingested carotenes (BCO1) and xanthophylls (BCO2) to apocarotenoids (9), which are eventually metabolized and excreted. This is in contrast to the primate BCO2’s poor cleavage activity which enhances xanthophyll uptake into many tissues. Most carotenoid delivery in humans is relatively nonspecific, and tissues such as skin and fat generally reflect blood carotenoid profiles. On the other hand, a few tissues take up L and Z with high affinity and specificity. Most notably, the ocular tissues take up L and Z from LDL and HDL via scavenger receptors of the retinal pigment epithelium (RPE) (10, 11). Once in the RPE, the retinoid isomerase (RPE65) enzyme converts some L to MZ through a C=C double-bond shift from the 5′-6′ position to the 4′-5′ position (12). While the RPE has a reasonably diverse array of carotenoids, only L, Z, and MZ are delivered to the retina, presumably via interphotoreceptor retinoid binding protein (13).

**Fig. 3:**
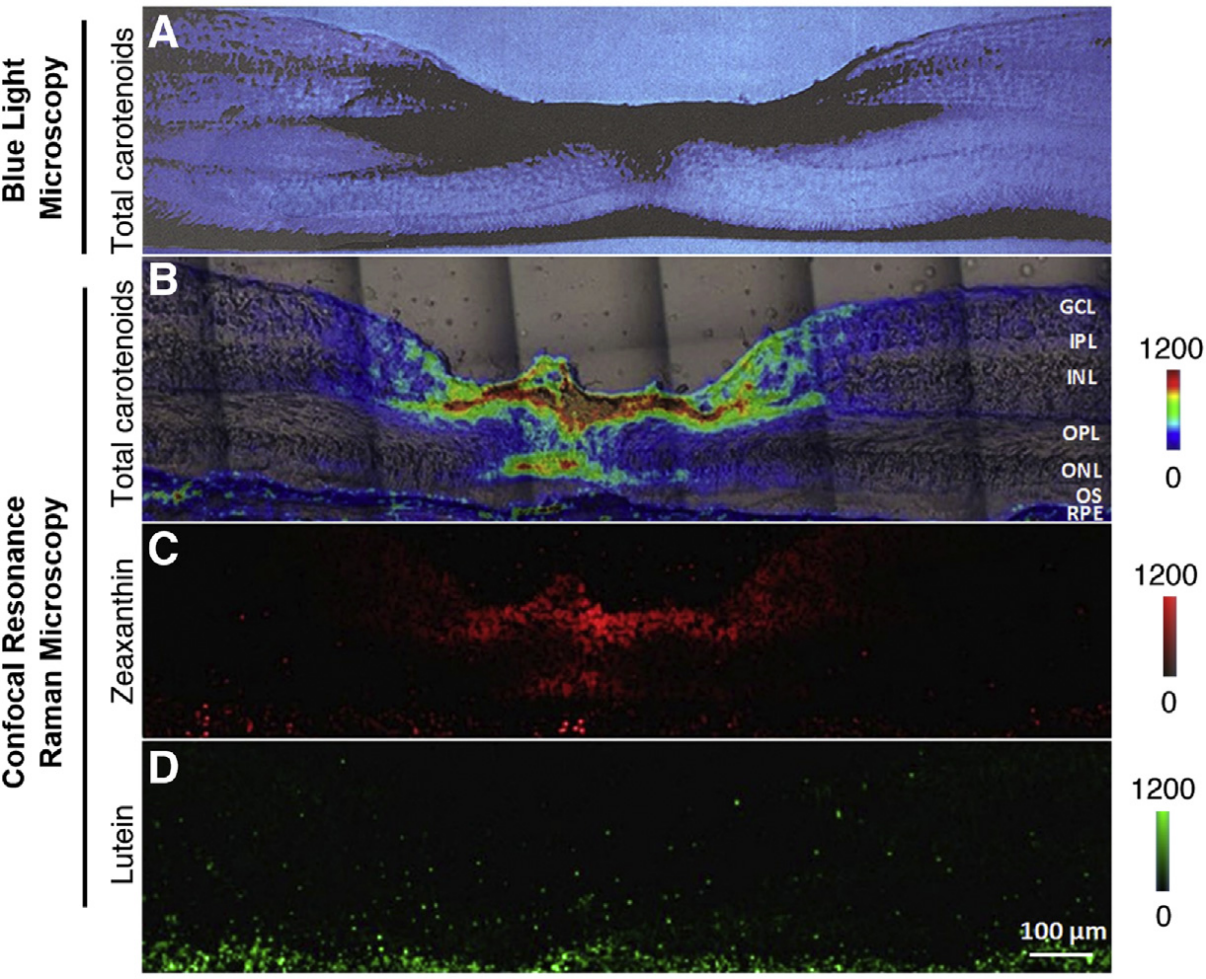
Comparison of images of the MP carotenoids in monkey and human foveal sections using blue light absorption or confocal RRS. A: A cross-section of a monkey’s fovea shows that the yellow MP appears dark when illuminated with blue light. This image was originally prepared by D. Max Snodderly, PhD. in 1984 and is reprinted with permission from Webvision (http://webvision.med.utah.edu/). B: An RRS image of total macular carotenoids (L+Z+MZ) is overlaid over a light microscope image of the human fovea that provided the RRS image. C: An RRS intensity map of human foveal Z (Z+MZ) generated using classical least squares fitting demonstrates that Z+MZ accounts for the vast majority of the foveal MP. D: An RRS intensity map of L generated using classical least squares fitting shows that L is diffusely distributed across the human fovea at much lower concentrations relative to Z+MZ. For details on how the RRS images were prepared, see (19). GCL, ganglion cell layer; IPL, inner plexiform layer; INL, inner nuclear layer; OPL, outer plexiform layer; ONL, outer nuclear layer; OS, outer segments.

Once in the retina, the MP carotenoids are distributed and stabilized through noncovalent associations with specific high-affinity binding proteins. In the case of L, the retina’s binding protein is steroidogenic acute regulatory domain protein 3 (StARD3), the human homolog of the silkworm’s L binding protein (14). For Z and MZ, glutathione S-transferase P1 (GSTP1) is the retina’s binding protein (15). The importance of GSTP1 as a physiologically relevant macular carotenoid binding protein has been confirmed by its recognition that genetic variants in GSTP1 are determinants of MP optical density (16). In the bloodstream, the L:Z:MZ ratio is about 3:1:0, similar to the diet, while in the peripheral retina, the ratio is 2:1:0.5 (17, 18). In the macula lutea (the yellow carotenoid-rich spot centered at the fovea centralis, the cone-abundant region of the primate retina responsible for high acuity color vision), the concentration of MP abruptly rises 100-fold to greater than 1 mM, and the L:Z:MZ ratio changes to 1:1:1 (17). This reported 1:1:1 ratio is based on relatively low-resolution HPLC methods, which typically require tissue punches several millimeters in diameter. More recently, we have separately imaged L and Z+MZ at high-resolution in human donor foveal sections using a confocal resonance Raman microscope, and we now compare it to the classic blue light microscopic image prepared by Snodderly in 1984 (Fig. 3) (19, 20). We found that the Z+MZ:L ratio can be greater than 9:1 at the foveal center. Moreover, we could clearly localize the foveal MP to the outer plexiform (Henle fiber) layer and the inner plexiform layer, with axial extension from the inner limiting membrane to the outer limiting membrane. On the other hand, the MP carotenoids were below the limit of detection in the photoreceptor outer segments even at the center of the fovea. Although we have not yet achieved cellular resolution with this imaging technique, our observed foveal MP distribution is highly consistent with the Müller cell cone of the fovea centralis described by Gass (21), but we cannot yet rule out cone axon localization as well.

Physiologically, the MP carotenoids are thought to be concentrated in the primate fovea for several reasons (1, 22, 23). which will be covered in more detail in the following sections. First, these intensely yellow compounds absorb blue light very efficiently and may be able to prevent long-term photo-oxidative damage induced by the most energetic light that we routinely encounter. Similarly, filtration of short-wavelength visible light may improve visual performance through a variety of mechanisms. Second, the MP carotenoids are efficient antioxidants that are deposited at high concentrations in a region of the body that is exposed to high levels of oxygen and visible light known to generate abundant reactive oxygen species and oxidative stress (24, 25). Finally, because the primate fovea and the MP are so intimately associated throughout the lifespan, there is the possibility that the MP carotenoids may promote normal foveal and visual development and even combat blinding diseases of childhood, such as retinopathy of prematurity (ROP) (26).

## The MP carotenoids and age-related retinal diseases

### Age-related macular degeneration

Age-related macular degeneration (AMD) is the first ocular disease to be conclusively linked to the MP carotenoids (27). This common cause of irreversible blindness in the elderly is due to a number of modifiable and nonmodifiable risk factors including aging, heredity, smoking, excessive light exposure, and nutrition. In the early and intermediate stages, there are deposits of oxidized lipids and proteins underneath the RPE known as drusen, thickening of Bruch’s membrane under the RPE, and increased levels of RPE lipofuscin, but visual function may still be near normal. In the advanced exudative or “wet” form of AMD, abnormal new vessels can infiltrate Bruch’s membrane or proliferate beneath the RPE, within the subretinal space, and even enter the retina itself. This neovascular tissue can leak fluid and blood and cause irreversible macular scarring and blindness if left untreated (27). Intravitreal injections of anti-vascular endothelial growth factor (anti-VEGF) compounds can combat wet AMD, but they may require long-term monthly in-office administrations of medications that are often very expensive and not always effective. In the advanced dry stage of AMD, large patches of RPE and retina can die away in the macula, leaving sharply demarcated patches known as geographic atrophy that will cause central visual loss if the fovea is affected. Currently, there is no effective treatment to reverse or slow down dry AMD, but several complement inhibitors and other medications are in clinical trials.

Due to the challenges of treating AMD, there has long been an interest in whether nutritional interventions could prevent or slow the progression of the disease, especially because there was a growing recognition that AMD was in part a disorder associated with oxidative stress. The original Age-Related Eye Disease Study (AREDS) utilized the best nutritional knowledge of the 1980s and tested the efficacy of zinc, vitamin C, vitamin E, and β-carotene and reported a statistically significant 25% reduction of the incidence of advanced AMD over a 5 year period with the full AREDS formulation versus placebo (28). While the AREDS was a major public health advance, there was recognition that the β-carotene component was problematic for a variety of reasons. First, there was much greater knowledge that ocular carotenoids are not all the same. L and Z had become widely known at that point to be specifically concentrated in the retina, while β-carotene was found in no more than trace amounts (17). Epidemiology studies were starting to show that high blood levels and high dietary consumption of L and Z were associated with decreased risk of advanced AMD, while β-carotene was not (29, 30). MP measurements in living human eyes showed that high MP levels were associated with lower relative risk of AMD and that MP levels could be raised through diet or supplementation (31). Finally, multiple studies were showing that β-carotene administered to smokers at the AREDS dose increased risk of lung cancer (32). Thus, the AREDS2 study was initiated in 2006 to test whether 10 mg/day of L and 2 mg/day of Z (±1,000 mg/day of omega-3 fatty acids derived from fish oil) would be an acceptable substitute for the β-carotene of the original AREDS formula. Although the AREDS2 study did not reach its ambitious primary endpoint of an additional 25% reduction in AMD risk relative to the original formulation (33), secondary analysis showed that 10 mg/day of L and 2 mg/day of Z was an acceptable substitute for 15 mg/day of β-carotene with a better safety profile (34), especially in smokers. Since that time, the AREDS2 dose of L and Z has been the standard-of-care for individuals with intermediate or advanced AMD in one or both eyes. It is less certain whether supplementation with L and Z would be helpful for the “worried well” with early or no signs of AMD, as such studies would be prohibitively large and prolonged, but there are several epidemiological studies that indicate that diets rich in L and Z are protective against AMD (30, 35, 36). Such diets should be especially encouraged to individuals with low MP or a strong genetic risk for AMD.

### Macular telangiectasia type 2

Macular telangiectasia type 2 (MacTel) is a much less common macular disorder that typically presents after age 40, which is associated with abnormal levels and distributions of MP (37). It causes moderate visual loss and makes reading particularly challenging due to paracentral scotomas. Optical coherence tomography reveals cavitations in the inner and outer retina near the fovea that can eventually collapse, leaving thinned and dysfunctional retina. In later stages, telangiectatic vessels appear temporal to the fovea and may eventually evolve to choroidal neovascularization. Although once considered a sporadic disease, we have demonstrated that the disease actually has a significant underlying genetic component because nearly 20% of first-degree relatives may be affected as well (38). One such Utah family with multiple affected family members was key to the discovery of the first MacTel gene, serine palmitoyltransferase long chain base subunit 1 (SPTLC1) (39). There is no effective treatment for MacTel, but a phase 3 clinical trial of an intravitreal implant that secretes ciliary neurotrophic factor is in progress (40, 41).

**Fig. 4:**
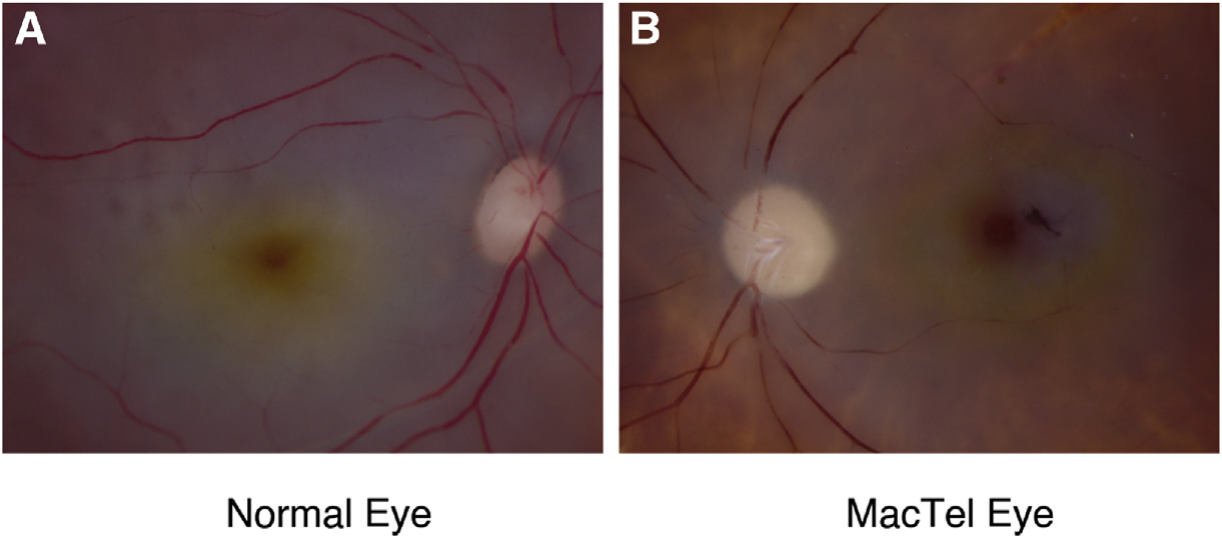
Comparison of MP distributions of a normal eye versus a MacTel eye. In a normal eye, the macular carotenoid pigment is a central yellow spot. In a MacTel eye, the macular carotenoid pigment redistributes into a yellow ring with no carotenoids present at the center of the macula. Images courtesy of Gregory Hageman, PhD.

In 2008, European researchers doing autofluorescence imaging of the retina on MacTel patients noticed unexpected abnormalities of the MP. Instead of a normal central peak of the MP, there was either complete absence of the MP or else a redistribution into a 5°-radius ring at the edge of the MacTel area (42, 43). This MP ring could also be observed in postmortem MacTel eyes (Fig. 4), and histopathology suggested that the central MP loss could be due to profound disruption of the foveal Müller glial cells. The aberrant MP ring formation could be due to binding protein redistribution, but this hypothesis has not yet been proven. Loss of central MP could lead to poor visual performance (see next section) beyond the damage to the outer retina observed by optical coherence tomography, so clinical interventional trials with L or Z have been conducted to try to fill in the missing foveal carotenoids in MacTel patients and potentially recover some lost visual function. High-dose supplementation with L or Z did not restore the central MP in any MacTel patients, but many of them showed enhanced MP rings or even carotenoid crystallization (44, 45).

## The MP carotenoids and visual performance

Although altered levels and distributions of MP are involved in the pathogenesis of AMD and MacTel, such diseases occur late enough in life that they cannot explain the evolutionary pressure that has selected for the near universal presence of the MP in humans and other primates. A number of researchers have suggested that high levels of MP confer visual performance advantages (46, 47, 48), presumably in a direct manner due to the MP carotenoids’ blue light-filtering properties or more indirectly through improved antioxidant status or enhanced neural processing. Indeed, another class of animals known for excellent visual performance, birds, actively concentrate a variety of carotenoids including L, Z, and MZ in oil droplets in their photoreceptor cells (49). Multiple groups have evaluated MP levels and MP supplementation in relation to various measures of visual performance, such as visual acuity, contrast sensitivity, and glare recovery, in both normal and AMD retinas (50, 51, 52). Although the functional improvements were small, they were often reproducible and statistically significant. Even if imperceptible to the average human, such performance enhancements could confer a competitive advantage in military, sports, or hunting activities. Interestingly, when the major xanthophyll carotenoid cleavage enzyme (BCO2) is knocked out in mice and they are then fed L or Z, the ensuing retinal deposition of carotenoids enhances their visual acuity and contrast sensitivity as measured by their optokinetic response in an OptoMotry® virtual rotating drum apparatus (53).

## The MP carotenoids in early life

### Physiology of the MP during ocular development

Autopsy studies of fetal and infant eyes have shown that MP carotenoids are detectable in the human retina as early as 20 weeks of gestation (54). This presence during a phase of life with no need for high acuity vision implies a physiological role in development of the fovea and other uniquely primate retinal structures. Moreover, it is intriguing that ocular albinism characteristically has both no foveal depression and minimal MP (55, 56, 57), but it is not yet known which one is the root cause of the developmental abnormality. Other animals that have high levels of ocular carotenoids, such as chickens, likewise deliver L and Z from the yolk to the eye prior to emergence to the outside world. Our laboratory reported that L and Z are detectable in the chicken retina and RPE/choroid by embryonic day 15 (E15), and steadily rise until right before hatching on E21 (58). MZ, which is not normally present in the egg yolk, initially appears in the RPE/choroid at E17 by way of enzymatic conversion from L by the RPE65 enzyme (12) and is then deposited in the retina a few days later.

### Measurement of MP in children

Further studies on MP physiology in children have been hampered by the unique challenges of carotenoid measurement in young individuals. Obviously, donor eyes of fetuses and infants are rarely available, and HPLC, although chemically specific, has inherently low spatial resolution due to the physical limits of tissue dissection. The most common techniques to measure MP in adults, such as heterochromatic flicker photometry and autofluorescence attenuation, are generally not possible in children younger than 7 years old due to inability to perform psychophysical tasks reliably and due to low levels of RPE lipofuscin, respectively (59, 60). On the other hand, we found that blue light reflectometry could image MP even better in infants than adults because of their very clear ocular media and the availability of video-based clinical instruments specifically designed to image the infant eye, such as the RetCam® (Natus Medical, Pleasanton, CA). By removing the fluorescein angiography blue light barrier filter in the light collection optics and by using its built-in blue light source, we could readily measure MP levels and distributions using single-wavelength reflectometry with the RetCam®, and we found that MP is detectable at birth and steadily rises to near adult levels by age 7 (61). A follow-up study by colleagues in Japan using the same technique demonstrated that MP was also measurable in premature infants (62).

### Carotenoid physiology during and after pregnancy

Several studies have shown that the mother actively and preferentially transports L and Z to her child via the placenta (53, 63), especially during the third trimester. We studied this further through a cross-sectional study of the ocular and systemic carotenoid status of newborns and their mothers. We measured serum carotenoids of the mother and child by HPLC and their total skin carotenoids noninvasively with resonance Raman spectroscopy (RRS) and then compared these values to MP levels measured in the babies with the RetCam® (64). We found that there were many statistically significant correlations between the mother’s and her child’s carotenoid status and that a high maternal serum Z level was the main driver of these correlations. We also noted that maternal serum and skin carotenoid levels were unexpectedly low relative to our normative database, implying that the mothers may have become carotenoid depleted during pregnancy. After delivery, premature infants can rapidly become carotenoid depleted due to severe oxidative stress and the fact that premature infant nutritional formulations typically do not include any carotenoids (65). Additional postnatal L and Z transfer to the full-term child can continue through breast milk or by L- and Z-fortified infant formulas.

### The L-ZIP study

Based on the active transfer of L and Z from mother to child during pregnancy and the potential depletion of maternal carotenoid stores, we asked why L and Z are so rarely incorporated into prenatal vitamins. Only Abbott Nutrition’s Similac Prenatal Vitamins® (Abbott Laboratories, Columbus, OH) has ever been marketed as a carotenoid-fortified maternal supplement in the United States (6 mg/day of L derived from marigold flowers), and it was withdrawn from the market in 2018 due to poor sales. We still felt that L and Z supplementation during pregnancy could have great benefits for both the mother and baby, but supportive level 1 clinical trial data were lacking to drive its adoption by obstetricians and their patients. We therefore initiated our own clinical trial.

**Fig. 5:**
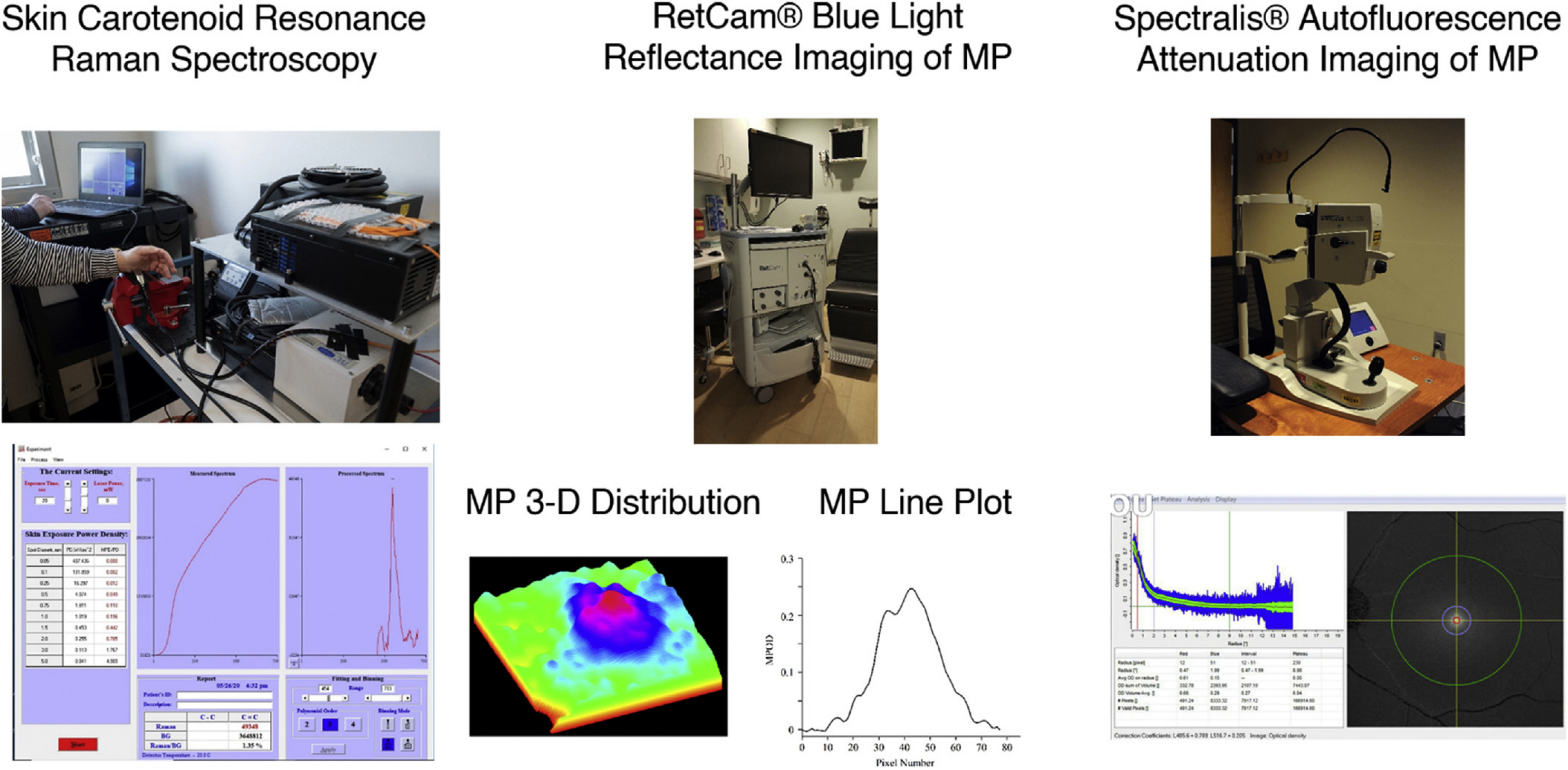
Three methods for carotenoid status assessment in the L-ZIP study. The first column shows our custom-built skin RRS device. Adults are measured by projecting the blue laser onto the palm of their hand. Babies are measured on the sole of their foot. A screenshot of the instrument’s output is shown below. The middle column is a Natus RetCam® 3, a clinical instrument designed to image the retina of a baby’s eye. We use its blue light source for reflectance imaging of the retina. Below are processed images of a baby’s MP as a 3D map or a line plot. The right column is a Heidelberg Engineering Spectralis®, a multimodal clinical instrument for imaging the human retina. Shown below is a screenshot of a human’s MP distribution as a radial plot with peak carotenoid at the fovea (0°) or as a 2D density plot with the MP displayed as a white spot centered at the fovea.

The Lutein and Zeaxanthin in Pregnancy (L-ZIP) study is a single-site randomized placebo-controlled double-masked clinical trial of 10 mg/day of L and 2 mg/day of Z versus a control gel cap of just the safflower oil vehicle (clinical trial NCT03750968 registered at https://clinicaltrials.gov/). We chose an AREDS2 dose of L and Z because of its established safety and availability and the fact that it contains substantial amounts of both L and Z relative to the average American diet. Mothers start active or control investigational product based on a 1:1 randomized allocation before the end of their first trimester in addition to their standard-of-care prenatal vitamins, which have no added carotenoids. Our primary outcome measures are the mother’s carotenoid status at the beginning versus the end of pregnancy measured in the serum (HPLC), skin (RRS (a picture of our instrument and its readout are shown in Fig. 5), and macula [autofluorescence imaging of the MP with a Spectralis® (Heidelberg Engineering, Heidelberg, Germany)]. We hypothesize that prenatal carotenoid supplementation can prevent maternal depletion of carotenoids that will occur during pregnancy. Our secondary outcome measures are assessments of the newborn infant’s carotenoid status in cord blood (HPLC), placenta (HPLC), skin (RRS), and macula (RetCam® blue-light reflectance imaging). We hypothesize that prenatal carotenoid supplementation will enhance the newborn infant’s ocular and systemic carotenoid status. Exploratory outcomes include changes in maternal visual acuity during pregnancy and infant foveal structure at birth (optical coherence tomography). As of this writing, 16 of 60 potential subjects have enrolled in the study, and the investigational supplement and control gelcaps have been well tolerated.

### Frontiers in prenatal and postnatal supplementation

The L-ZIP study will provide necessary data to design and power future larger scale prospective clinical trials with further optimized formulations to determine whether prenatal and postnatal carotenoid supplementation can have beneficial effects on maternal visual function and on infant visual development in normal and high-risk pregnancies. We plan to reassess the enrolled infants in future years to determine whether long-term functional benefits are detectable in children of mothers who received the active prenatal supplement versus control. The results may also provide evidence-based support to guide policy decisions about prenatal nutritional recommendations to enhance maternal and infant carotenoid status, especially in regions of the world at risk for malnutrition. Although carotenoid supplementation to premature infants did not prevent ROP in previous clinical trials (66), the dose may have been too low and too late. We suggest that maternal prenatal supplementation with L and Z might be more efficacious against ROP and should be examined in a future clinical trial.

## Conclusions

While the yellow MP of the human fovea has been known to anatomists for centuries, the last few decades have seen considerable advancement in our understanding of the fundamental roles of L, Z, and MZ in human macular health and disease. It is fascinating that primates have repurposed photoprotective pigments and even some of their binding proteins from plants and insects to protect and enhance visual acuity throughout the lifespan. These natural products are now well known to optometrists, ophthalmologists, and their patients and are widely recommended as essential components of the AREDS2 formulation for individuals at risk for visual loss from AMD. From a clinical standpoint, there are still many questions to be answered with regard to optimal supplement formulations and preferred methods to monitor ocular and systemic carotenoid status. In the basic science realm, there is still much to learn about carotenoid transport and metabolism and about the biochemical and biophysical mechanisms underlying the photoprotective and antioxidant effects of the MP. In our opinion, the potential for addition of L and Z to prenatal and postnatal supplements is one of the most exciting new research avenues to explore. We hope that this overview has provided the reader with sufficient insights to spark additional progress on these macula-specific lipids.

## Conflict of interest

Paul S. Bernstein and the University of Utah hold patents on the use of RRS to measure carotenoids in the eye and other tissues.
